# Uncovering Hidden Framings in Dark Triad Self-Ratings: What Frames-of-Reference Do People Use When Responding to Generic Dark Triad Items?

**DOI:** 10.1177/10731911231220357

**Published:** 2024-01-29

**Authors:** Julian Schulze, Manuel Heinrich, Jan-Philipp Freudenstein, Philipp Schäpers, Stefan Krumm

**Affiliations:** 1 Freie Universität Berlin, Germany; 2 University of Münster, Germany

**Keywords:** Dark Triad, frame-of-reference, symmetry principle, context, interpersonal, situation, personality

## Abstract

In typical Dark Triad (DT) questionnaires, generic items oftentimes refer to “others” or “people” in general. Hence, respondents have to mentally aggregate their behavior across several kinds of “others” (e.g., work colleagues, family members, and friends). It remains unknown if individuals consider different kinds of interaction partners equally or if their self-reports contain “hidden” interaction partner-specific tendencies. To shed light on this issue, we assessed generic and contextualized DT items (referring to family, friends, work, and strangers; *N* = 814 from the general population). The correlated trait-correlated (method − 1) model was used to investigate preregistered research questions. On average, generic DT items showed the strongest association with work-contextualized DT items and the weakest association with family-contextualized DT items. However, the associations varied considerably across DT items and traits. In sum, our results suggest that hidden framings exist in some DT items, which may impact their ability to predict relevant criteria due to contextual (a)symmetries. The generalizability of the findings to other DT instruments, items, and participant groups should be examined in future research.

Research on the Dark Triad (DT) of personality—Machiavellianism, narcissism, and psychopathy—has grown tremendously over time (Muris et al., 2017). Several reviews and meta-analyses summarized the relationships of the DT with important life outcomes such as well-being (Muris et al., 2017), job performance, and creativity (LeBreton et al., 2018), illustrating their cross-disciplinary relevance. Recently, studies on person-situation interactions emerged as another growing research branch in the DT literature (e.g., Grover & Furnham, 2021). In particular, *interpersonal situations* are considered crucial for the expression of DT traits (e.g., Rauthmann, 2012). The focus on interpersonal contexts is also mirrored in many assessment instruments of the DT. For instance, most items in the prominent Short Dark Triad questionnaire (Jones & Paulhus, 2014) focus on behavior directed toward “others” or “people.”

When responding to personality items, it is usually assumed that individuals form a mental aggregate of their relevant experiences (see Beckmann et al., 2010; Fiske, 1986; Lewandowski & Nardone, 2012; Wood, 2007). Although there is some controversy over whether individuals need to recollect experiences from autobiographical memory or whether they rely on “abstract summary traits” that have formed over their lifetime (Klein et al., 2002, p. 311), the different views converge on the idea that responding to personality items require some form of aggregate judgment. Given the emphasis on interpersonal interactions in the DT literature and in DT assessment tools, an important yet unanswered question is which interaction partners individuals integrate when responding to generic DT items. Terms such as “others” or “people” are broad in meaning and can relate to a plethora of individuals. Therefore, DT items that contain implicit or explicit context cues potentially permit individuals to use idiosyncratic contextual framings (Lievens et al., 2008; Schulze et al., 2021). As a response to this “black box” of generic DT judgments, the current study examines if individuals (from the general population) form an aggregate of DT behaviors that gives equal weight to a variety of interaction partners or if individuals refer to certain kinds of interaction partners more readily compared with others (e.g., make stronger reference to strangers compared with family members). In other words, we investigate whether “hidden” interpersonal framings exist in DT items. In addition, this study examines whether such hidden framings bear consequences for the predictive power of the DT (i.e., in explaining contextualized interpersonal deviance as a criterion variable). Thus, our study contributes to DT theory and, at the same time, highlights potential practical implications of our findings.

## Theoretical Background

Generic personality items are common in psychological research and require that respondents make summary judgments across time and contexts (e.g., Lewandowski & Nardone, 2012). As Fiske (1986) notes (p. 40), “we leave this summarizing task to our subjects, permitting them to do it as they wish.” DT assessment instruments are no exception to this practice. Typically, DT indicators are based on a *behavior* aspect (e.g., manipulating, getting revenge, being mean; see Jones & Paulhus, 2014) and a *situation* aspect (i.e., what contexts need to be considered by respondents). A considerable number of DT items focus on behavior toward “others” or “people” in general (Dirty Dozen, Jonason & Webster, 2010; Short Dark Triad, Jones & Paulhus, 2014). For instance, we counted that 16 of the 27 items of the Short Dark Triad questionnaire (SD3; Jones & Paulhus, 2014) include explicit context cues such as “others” or “people.” Notably, the wording of many other DT items implicitly suggests an interpersonal context even without explicit cues (see also De Raad, 2005). For instance, the wording “It’s not wise to tell your secrets” (Jones & Paulhus, 2014, p. 38) is arguably understood as “It’s not wise to tell your secrets *to others*.” In conclusion, many DT items require the respondent to mentally aggregate behavior over many interaction partners (e.g., others or people in general).^1^

### Hidden Interpersonal Framings in the DT

**Figure 1.**
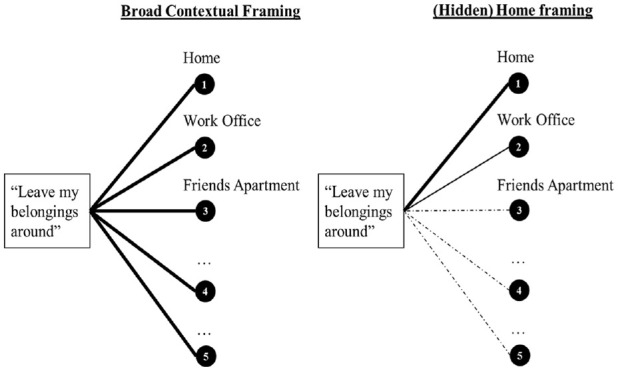
Visualization of Contextual Framings in Generic Personality Items. *Note*. Left panel: Generic scores reflect all contexts equally well. Right panel: Generic scores reflect different contexts differently, whereby the item contains a particularly strong “hidden” home-context framing; [. . .] indicate additional contexts; “Leave my belongings around” is an orderliness indicator from the International Personality Item Pool (Johnson, 2014, p. 82).

Recently, Schulze et al. (2021) reviewed the literature on the symmetry principle (Wittmann, 1988) and the frame-of-reference effect (Lievens et al., 2008) and presented an integrative model. They concluded that generic personality items might contain “hidden” contextual framings. That is, even though often formulated in a generic way, items in personality scales may nevertheless differ in contextual specificity. This idea is depicted in Figure 1. For example, the item “Leave my belongings around” (an indicator of the personality facet ‘orderliness’; Johnson, 2014, p. 82) may potentially be related to a variety of contexts (e.g., work office, home). However, respondents may not mentally aggregate their behavior across these different contexts with equal weight (Figure 1, left panel) but may have, for instance, a stronger focus on the home context compared with other contexts (Figure 1, right panel). This weighting might be due to the prototypical character of the home context of “orderliness,” contributing to a hidden contextual “home” framing (see also situation/behavior prototype literature, Cantor et al., 1982; Mischel & Peake, 1982).

We argue that the notion of hidden framings, as proposed by Schulze et al. (2021), can be applied to DT measurement. Rather than representing DT behavior toward different interaction partners equally well, generic DT items may contain hidden interpersonal framings toward specific groups of “others” or “people.”Tett et al. (2021, p. 201) reasoned that “traits are engaged when called on by the right type of situation” and assumed that situations contain “triggering” elements for the expression of certain personality traits. Trait-relevant cues for the expression of DT behavior may frequently be present in certain kinds of interpersonal situations but less often in others. For example, competition is a prevalent workplace characteristic that may have the potential to activate DT-related behavior frequently (see Castille et al., 2017, for a study on Machiavellianism). Although competition may also play a role in interpersonal situations with friends or family, these relationships are often characterized by high levels of closeness (Berscheid et al., 1989). Such counteracting interpersonal features could potentially lead to less frequent expression of DT behavior. Ultimately, the link between specific interpersonal contexts and the frequency of expressions of the DT could potentially shape the mental aggregates formed while responding to generic DT judgments. Consequently, respondents may use interpersonal contexts in which the DT is expressed frequently as “prototypical” reference contexts in the response process.

### Potential Consequences of Hidden Framings

Understanding and revealing potential hidden framings in generic DT items is important for several reasons. First, hidden framings in DT items may not balance out completely when aggregating items to scale scores. So, not only DT items themselves but also DT scale scores may contain a hidden framing, thereby impacting their construct-related validity. Second, many studies use DT scale scores to predict criteria from specific interpersonal contexts such as romantic relationships (e.g., intimacy, passion, Ali & Chamorro-Premuzic, 2010; relationship satisfaction, Rentzsch et al., 2021), friendships (e.g., conflict, Wehner & Ziegler, 2023), situations with strangers (e.g., popularity, Back et al., 2010), or situations with work colleagues (e.g., counterproductive work behavior, DeShong et al., 2015). However, linking generic ratings that contain hidden framings to contextualized criteria may impact contextual symmetry (Schulze et al., 2021; Wittmann, 1988; see also Murtha et al., 1996): In the case of a hidden contextual framing in the DT trait that does not match to the context of the criterion (= high hidden contextual *asymmetry*), a researcher unaware of hidden framings could potentially underestimate the predictive power of a personality trait. It is also possible that a researcher overestimates the predictive power of a trait if the trait under investigation has a hidden framing that matches particularly well to the criterion framing (= high hidden contextual *symmetry*) while other personality predictor variables do not match well with the criterion framing. So, the value of the symmetrical trait in predicting criterion variance may be overestimated relatively to the other traits.

**Figure 2.**
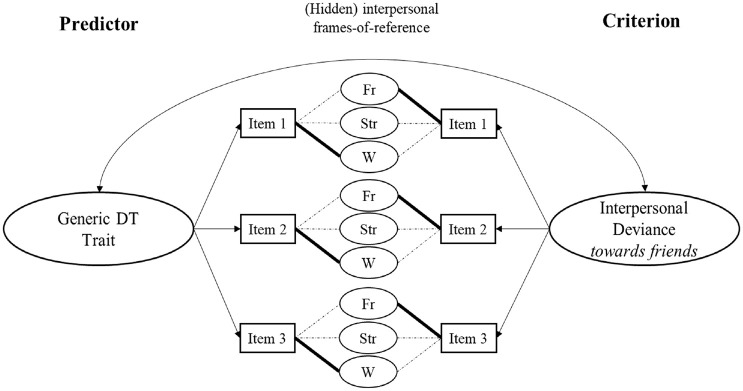
Visualization of the Potential Consequences of Hidden Framings for the Predictive Power of DT Traits. *Note*. If generic DT items do (not) represent particular contexts relevant for the criterion, contextual (a)symmetries may emerge, boosting (lowering) the predictive power of the trait. In the illustrated case, generic DT items do not appropriately represent “dark” behaviors toward friends. This may result in a contextual mismatch with the friends-contextualized criterion variable. Solid black lines indicate a strong (hidden) framing to the particular context. Dashed thin lines indicate weaker (hidden) framing to a particular context. DT = Dark Triad; Fr = Friends framing; Str = Strangers framing; W = Work framing.

To illustrate further, Figure 2 shows three generic DT items (left side of the figure) and three interpersonal deviance items contextualized toward *friends* as criterion measures (right side of the figure). Note that interpersonal deviance is a criterion frequently related to facets of the DT (DeShong et al., 2015; Min et al., 2019; Witt & Donnellan, 2008). In the illustrated case, the hidden work-related framing of the DT aggregate score (left side of Figure 2) is partly inconsistent with the contextualized interpersonal deviance criterion (i.e., focusing on deviant behavior toward friends), so the trait’s predictive value might be underestimated. Importantly, despite this particular illustration of underestimating predictive power, hidden framings may also contribute to an overestimation of the predictive power of traits, or may have no effect at all, depending on the particular case under investigation. Therefore, understanding which contextual framings individuals apply is important to accurately estimate associations between the DT and criterion variables.

### Preregistered Research Questions

We preregistered three research questions to investigate hidden framings on the item-level (see Open Science Framework [OSF] link for the preregistration PDF):

**Research Question 1 (RQ1):** First, we will investigate if the same generic DT traits (i.e., psychopathy, Machiavellianism, and narcissism) show differential consistency to differently contextualized equivalents (family, friends, colleagues, strangers . . .) on the item level.^2^

Thus, RQ1 investigates how much variance a particular *generic* DT item can explain in *contextualized* versions of the very same item. Focusing on each context individually and on differences in the consistency coefficient between the contexts allows us to identify interaction partner-specific tendencies (hidden framings) in the generic items: If the generic item correlates strongly (high consistency) with one context (e.g., friends) but considerably less so (low consistency) with another context (e.g., strangers), this could potentially speak for such an interaction partner-specific tendency. As there is a growing interest in the item-specific effects of personality measures (McCrae & Mõttus, 2019), we apply an indicator-specific perspective. Although RQ1 focuses on each item of a DT trait in isolation, we will advance this perspective by comparing the explained variance by the generic items *across* the three DT traits:

**Research Question 2 (RQ2):** Second, we will investigate if different generic DT traits (i.e., psychopathy, Machiavellianism, and narcissism) show differential patterns of consistency coefficients to their contextualized equivalents (family, friends, colleagues, strangers . . .) on the item level.

Third, we aimed at gaining additional insights as to how the effects of contextualization generalize across interpersonal contexts and DT traits:

**Research Question 3 (RQ3):** Finally, we will investigate how framing-specific method factors are intercorrelated within and across traits.

Finally, ancillary (exploratory) analyses with DT scale scores were conducted to examine whether item-level hidden framings may persist in aggregated DT scores and may potentially impact their predictive power. To address our RQs, we conducted a pilot and a main study.

## Pilot Study

The pilot study served two purposes: (a) To create context tags covering a reasonable spectrum of everyday interaction partners that could be used to contextualize DT items. (b) To identify a manageable set of generic DT items suitable for contextualization and use in our latent variable models of the main study. The supplemental material contains a detailed description of the pilot study. Code to replicate the analyses can be downloaded from the OSF project.

First, based on prior literature (e.g., Wrzus et al., 2016) and discussions in the research team, the following four context tags each relating to the situational domain of interpersonal associations as identified by Saucier et al. (2007) were agreed upon: (a) persons from the family (hereinafter referred to as “family”), (b) persons from the circle of friends (“friends”), (c) persons you work with (“work”), and (d) persons one hardly knows (“strangers”).

Subsequently, we used a publicly available dataset of a published study that used an *English* version of the SD3 questionnaire (https://osf.io/xey8h/; Vize et al., 2020). We used this data to prepare an item set for the main study. Note that although the pilot data were based on the English SD3 version, the main study, conducted in Germany, used the German version of the SD3 (see Malesza et al., 2019). At the time of data collection, we could not identify open data based on the German SD3 version comparable to the Vize et al. (2020) data. After screening out SD3 items not suitable for contextualization (6 of 27 SD3 items), we used an item selection procedure (bruteforce; Schultze, 2018) to construct a 15-item version of the SD3 suitable for our main study (five items per DT trait; see OSF materials for details).

## Main Study

### Method

#### Sample

Since limited resources were available to collect data (Lakens, 2022; see preregistration document), no more than *n* = 814 individuals could be sampled. We recruited our sample via a noncommercial German online panel for psychological research (comprising individuals from the general population; available through https://psyweb.uni-muenster.de). Several high-impact studies used data collected in this panel (e.g., Hirschfeld et al., 2014; Schäpers et al., 2021; Windscheid et al., 2016). The panel administration checked the quality of the study design and the suitability of the panelists concerning the requirements of the questionnaire (e.g., length and duration). As this is a noncommercial panel, it has less of a “superworker” problem (i.e., the top 5% of participants took ~40% of all studies; Robinson et al., 2019). The institutional ethics committee approved the study protocol (nr. 013.2021). Before accessing the survey, all participants provided informed consent.

The raw dataset consisted of 1,368 rows. We excluded 218 empty rows and subsequently deleted data of nine individuals who provided parts of the survey data twice (18 rows; this data cleaning step was not preregistered). Afterwards, we cleaned the data according to the preregistration. Specifically, we only considered individuals who (a) indicated having contact with all four interaction partner groups at least very rarely, (b) pursued gainful employment, (c) were at least 18 years old, and (d) provided complete survey data (this was true for a total of *n* = 831 cases). Next, individuals were excluded from the analyses if they either (e) stated that their data should not be used for analysis (*n* = 3) or (f) completed the survey in <5 min (*n* = 2). After excluding these cases, individuals who (g) got a wrong answer on more than two of five attention check items were also excluded (*n* = 12).^3^ This process resulted in a final sample size of *n* = 814 cases. Code and data to replicate the analyses can be downloaded from the OSF project.

The mean age of the respondents was 47.2 (*SD* = 12.3; range: 18–76 years). More women (~66.2%) than men (~33.4%) participated in the study (< 1% classified their gender as “other”). Most individuals reported a high degree of education (63.3% stated to hold a university degree or a university degree of applied sciences).

#### Measurement Instruments and Survey Design

First, participants reported demographics (gender, age, education) and responded to the 15 generic (original) SD3 items (*German* version, see Malesza et al., 2019). Afterward, they rated their frequency of contact with the four interaction groups (e.g., How frequently do you interact with members of your family, e.g., by phone, face-to-face, or chat?) and their employment status. Only respondents who indicated to be employed and to have contact with each of the four interaction partner groups at least very rarely were given access to the second part of the survey. This part consisted of the four contextualized DT versions (family, friends, work, and strangers; 60 items in total) presented in randomized block order. In addition, interpersonal deviance indicators (ID; adapted from Bennett & Robinson, 2000) were presented at the end of the survey in randomized block order (28 items in total).^4^ The ID items were also augmented with context tags referring to the four interaction partner groups (e.g., *I curse at persons from the family*) and serve as criterion variables in the ancillary analyses.

We assessed the contextualized DT items, employment status, and interaction frequency *after* the generic DT items to avoid priming toward a particular context (see preregistration for a detailed study flow). Following general recommendations (Chmielewski & Kucker, 2020; see also Meade & Craig, 2012 for this advice), we included six careless response indicators (five attention check items and one honesty statement).

#### Analysis Plan

**Figure 3.**
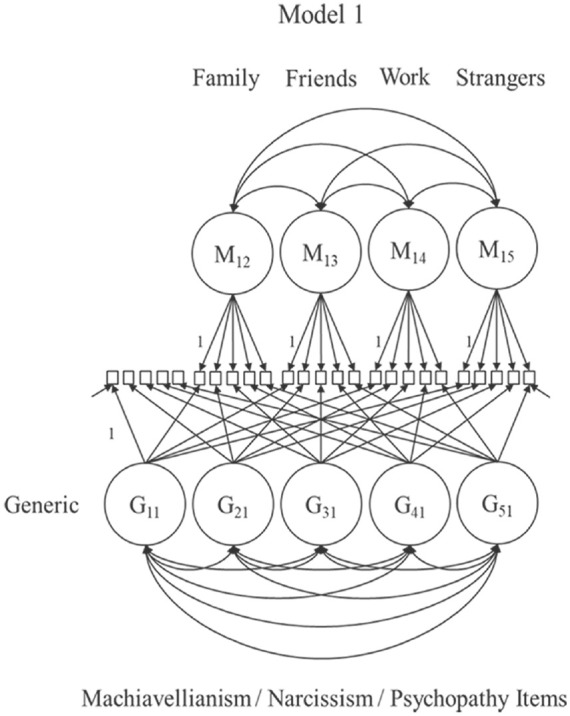
CT-C(M − 1) Model With Indicator-Specific Latent Variables (Eid et al., 2008). *Note*. This model was estimated three times (i.e., for Machiavellianism, narcissism, psychopathy). We identified each indicator-specific latent variable G_ij_ (i = number of the indicator within a Dark Triad trait j; j = the Dark Triad trait) by setting the loading of the item with the generic framing to 1. Correlations between indicator-specific latent variables are permitted. Method factors M_jm_ (m = the framing) are included for each nonreference framing (context) and identified by setting one of the contextualized item’s loadings to 1. Correlations between method factors are permitted, but correlations between indicator-specific latent variables and method factors are constraint to zero. No item labels and not all loading parameters and error variances depicted to avoid clutter. CT-C(M − 1) = correlated trait-correlated (method − 1); G = generic reference item; M = method factor.

**Figure 4.**
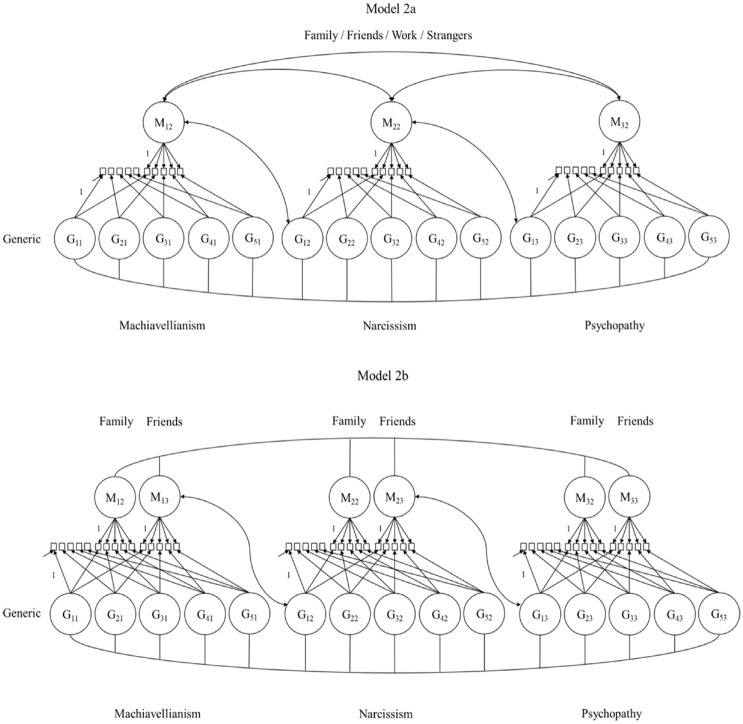
Model Complexity Was Increased Stepwise. *Note*. Models 2a to d include all generic Dark Triad items. Model 2a was fitted four times (each with a different method). Model 2b included method factors for family and friends contextualizations. This model was extended with work (Model 2c [not depicted]), and strangers method factors in final Model 2d (see Figure 5). No item labels and not all loading parameters, correlations, and error variances depicted to avoid clutter. In the process, narcissism item 3 was excluded from Models 2a to d due to a negative variance estimate. G = generic reference item; M = method factor

**Figure 5:**
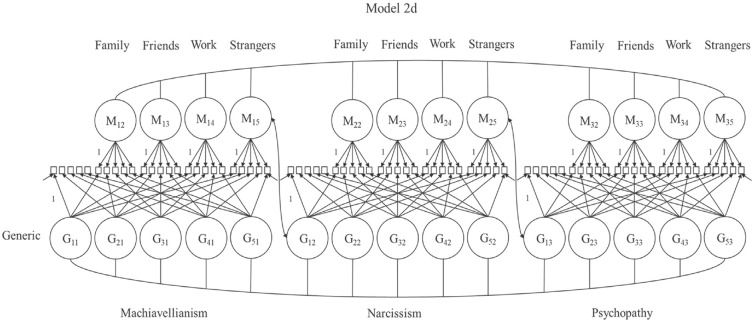
Final CT-C(M − 1) model with indicator-specific latent variables (see Eid et al., 2008, for details). *Note*. All generic reference items and all 12 method factors (for family, friends, work, and strangers per Dark Triad trait) were included in this final model step. No item labels and not all loading parameters, correlations, and error variances depicted to avoid clutter. In the process, narcissism item 3 was excluded from Models 2a to d due to a negative variance estimate. CT-C(M − 1) = correlated trait-correlated (method − 1); G = generic reference item; M = method factor.

A series of latent variable models with increasing complexity following a preregistered decision tree was estimated. The correlated trait-correlated (method − 1) model (CT-C[M − 1]) with indicator-specific latent variables by Eid et al. (2008) was the basis for all models (see Figures 3–5).

To illustrate the CT-C(M − 1) model, we refer to Figure 3 [Model 1]. This model includes all generic and contextualized items assessing the same DT trait (e.g., five generic psychopathy items + 20 contextualized psychopathy items). We estimated this model for each trait. The generic DT items load exclusively on the indicator-specific latent variables (G_ij_; i = number of the indicator within a Dark Triad trait j; j = the Dark Triad trait) and serve as reference items. All contextualized items load on one indicator-specific latent variable defined by the corresponding generic reference item and one method factor (M_jm_; m = the context). Items with the same context tag load on the same method factor. Method factors capture the true-score variance of the contextualized items not shared with the generic framing. Each model includes four method factors for family, friends, work, and strangers. Method and indicator-specific factors of the *same* DT trait are uncorrelated (Eid et al., 2008).

Using the models depicted in Figures 4 and 5, we increased the complexity in a stepwise manner. Contextual variants of Model 2a included *all* generic DT items but only one set of contextualized items. This model included one method factor per trait and was fitted four times, including family, friends, work, or strangers as framing. In Models 2b and d, we added the remaining contexts: Model 2b included method factors for family and friends. Model 2c added method factors for the work context (not depicted). Finally, Model 2d included all generic items and method factors for all contexts simultaneously.

The final CT-C(M − 1) model (Figure 5) decomposes the variance of the contextualized indicators into (a) variance shared with the corresponding generic DT indicator, (b) context-specific variance, and (c) measurement error. Based on this decomposition, we computed two important indices: the *consistency* and the *specificity* coefficient (see Eid et al., 2008 for formulas). Consistency describes the true-score variance in the contextualized item explained by the generic DT indicator. Specificity describes a context-specific part of the true-score variance of the contextualized DT item that the generic DT item cannot explain. If only *true-score* variance is considered, consistency and specificity sum up to 1 (Eid et al., 2003). A high consistency indicates that the generic indicators explain a large amount of reliable variance in the contextualized version and that individuals may apply this context implicitly while rating the generic form. A high specificity indicates that the context seems less represented by the generic rating (RQ1). We compared consistency and specificity coefficients descriptively across DT facets (RQ2). To allow for a better judgment of the preciseness of the estimates, we also computed 95% confidence intervals (CIs) for the consistency coefficients (based on 1,000 bootstrap draws using Mplus, Muthén & Muthén, 2017; not preregistered). Finally, correlations between method factors and correlations between indicator-specific latent variables were examined. High correlations between method factors across DT traits indicate that context effects generalize across DT traits (RQ3).

We assessed model fit and convergence at each step of the decision tree. Model fit was assessed using preregistered criteria (models with a CFI ≥ .90, RMSEA ≤ .08, and SRMR ≤ .10 were considered acceptable, see Schweizer, 2010). We used the maximum likelihood robust estimator to account for skewed data (MLR; West et al., 1995) and the R package “lavaan” (Rosseel, 2012; version 0.6-10) to fit all latent variable models. The next step of the decision tree was only conducted when no estimation problems emerged.

### Results

#### Latent Variable Models

All CT-C(M − 1) models had satisfactory model fit according to CFI, RMSEA, and SRMR (see Table 1). When fitting Models 2a including family method factors (and also Model 2a, including friends’ method factors; see Figure 4), the third narcissism item caused a nonpermissible solution (negative variance estimate for this item). The item (“I feel embarrassed if someone compliments me”, Jones & Paulhus, 2014, p. 38) was the only inversely worded one and showed low convergent validity with the other narcissism indicators (see supplements). To avoid nonpermissible solutions, the indicator was discarded from all subsequent models, and the family and friends’ Model 2a was re-estimated without the item. No further model modifications were needed.

**Table 1.** Model-Fit Indices for the Preregistered Correlated Trait-Correlated (Method − 1) Models With Indicator-Specific Latent Variables.

	χ^2^	*df*	*p*	CFI	RMSEA	SRMR
Models 1						
Machiavellianism + all context factors	538.595	239	<.001	.971	.041	.031
Narcissism + all context factors	497.193	239	<.001	.975	.039	.042
Psychopathy + all context factors	536.505	239	<.001	.968	.045	.036
Models 2a						
Dark Triad + family context factor^a^	501.014	214	<.001	.965	.040	.029
Dark Triad + friends context factor^a^	389.750	214	<.001	.979	.033	.022
Dark Triad + work context factor^a^	310.800	214	<.001	.990	.024	.018
Dark Triad + strangers context factor^a^	413.391	214	<.001	.977	.035	.021
Model 2b						
Dark Triad + family and friends context factors^a^	1,094.252	629	<.001	.969	.032	.041
Model 2c						
Dark Triad + family, friends, and work context factors^a^	2,230.858	1,231	<.001	.959	.033	.031
Model 2d						
Dark Triad + all context factors^a^	3,481.734	2,020	<.001	.954	.031	.037

*Note.* Solution based on Maximum Likelihood Robust estimator (MLR); χ^2^ = chi-square test statistic; *df* = degrees of freedom; CFI = comparative fit index; RMSEA = root mean square error of approximation; SRMR = standardized root mean square residual.

a Narcissism indicator 3 was excluded because of a negative variance estimate in the family and friends Models of Step 2a.

#### RQ1—Consistency Within DT Traits

RQ1 focused on the consistency coefficients within each DT trait (the explained variance in the contextualized items by the corresponding generic reference items). The parameter estimates from the final CT-C(M − 1) model (Model 2d) are given in Tables 2 to 4. The Tables highlight the consistency coefficients with their corresponding confidence intervals. In addition, Figures 3 to 5 visualize the estimated coefficients using barplots. To also provide an overall picture, we averaged the consistency coefficients for each context within a DT trait and computed a confidence interval around this average.

**Table 2.** Manifest Correlations and Variance Components Obtained From the Correlated Trait-Correlated (Method − 1) Model With Indicator-Specific Latent Variables for Machiavellianism Items (Model 2d—see Main Text and Figure 5 for Details).

Machiavellianism items	Observed variables			True-score variables	
	*M*	*SD*	Manifest correlation	Consistency [95% CI]	Specificity
Generic I1	2.64	1.10	—	1	
Family I1	2.14	1.08	.47	.59 [.49, .70]	.41
Friends I1	1.99	1.00	.57	.69 [.59, .78]	.31
Work I1	2.57	1.13	.64	.75 [.68, .82]	.25
Strangers I1	2.42	1.14	.54	.59 [.51, .69]	.41
Generic I2	2.38	1.02	—	1	
Family I2	1.87	0.99	.38	.56 [.46, .68]	.44
Friends I2	1.89	0.97	.45	.62 [.51, .72]	.38
Work I2	2.71	1.14	.61	.83 [.77, .90]	.17
Strangers I2	2.15	1.06	.50	.56 [.47, .68]	.44
Generic I3	2.28	1.10	—	1	
Family I3	1.60	0.88	.42	.36 [.28, .45]	.64
Friends I3	1.55	0.81	.49	.45 [.37, .54]	.55
Work I3	2.33	1.15	.68	.72 [.65, .79]	.28
Strangers I3	2.10	1.12	.58	.64 [.55, .73]	.36
Generic I4	2.22	1.02	—	1	
Family I4	1.59	0.84	.47	.58 [.47, .69]	.42
Friends I4	1.63	0.82	.58	.73 [.65, .81]	.27
Work I4	2.26	1.09	.59	.78 [.70, .86]	.22
Strangers I4	2.49	1.19	.54	.72 [.63, .81]	.28
Generic I5	4.02	0.78	—	1	
Family I5	3.17	1.18	.53	.90 [.86, .94]	.10
Friends I5	3.20	1.16	.59	.98 [.95, .99]	.02
Work I5	3.57	1.01	.64	.95 [.92, .98]	.05
Strangers I5	3.37	1.11	.58	.94 [.90, .97]	.06

*Note.* I = Item; Manifest Correlation = manifest correlation with corresponding generic item; CI = confidence interval based on 1,000 bootstrap draws.

**Table 3.** Manifest Correlations and Variance Components Obtained From the Correlated Trait-Correlated (Method − 1) Model With Indicator-Specific Latent Variables for Narcissism Items (Model 2d—see Main Text and Figure 5 for Details).

Narcissism items	Observed variables			True-score variables	
	*M*	*SD*	Manifest correlation	Consistency [95% CI]	Specificity
Generic I1	2.49	1.01	—	1	
Family I1	2.44	1.12	.60	.72 [.61, .90]	.28
Friends I1	2.41	1.06	.67	.80 [.66, .90]	.20
Work I1	2.58	1.08	.68	.81 [.69, .89]	.19
Strangers I1	2.13	0.99	.54	.59 [.46, .69]	.41
Generic I2	2.33	1.01	—	1	
Family I2	2.50	1.14	.48	.47 [.20, .62]	.53
Friends I2	2.43	1.09	.59	.70 [.49, .87]	.30
Work I2	2.34	1.06	.61	.73 [.57, .90]	.27
Strangers I2	1.89	0.98	.56	.66 [.54, .79]	.34
Generic I4	1.94	1.02	—	1	
Family I4	1.88	1.02	.58	.81 [.70, .93]	.19
Friends I4	1.98	1.04	.64	.92 [.83, .98]	.08
Work I4	1.77	0.92	.60	.91 [.82, .97]	.09
Strangers I4	1.79	0.99	.60	.78 [.66, .88]	.22
Generic I5	2.76	1.07	—	1	
Family I5	2.65	1.20	.53	.97 [.89, 1.00]	.03
Friends I5	2.50	1.14	.62	.98 [.92, 1.00]	.02
Work I5	2.95	1.19	.67	.95 [.88, .99]	.05
Strangers I5	2.64	1.21	.60	.92 [.84, .97]	.08

*Note.* I = Item; Manifest Correlation = manifest correlation with corresponding generic item; CI = confidence interval based on 1,000 bootstrap draws. Item 3 was excluded, see main text for details.

**Table 4.** Manifest Correlations and Variance Components Obtained From the Correlated Trait-Correlated (Method − 1) Model With Indicator-Specific Latent Variables for Psychopathy Items (Model 2d—see Main Text and Figure 5 for Details).

Psychopathy items	Observed variables			True-score variables	
	*M*	*SD*	Manifest correlation	Consistency [95% CIs]	Specificity
Generic I1	1.95	1.03	—	1	
Family I1	1.47	0.78	.43	.47 [.37, .58]	.53
Friends I1	1.40	0.68	.46	.50 [.40, .59]	.50
Work I1	1.81	1.00	.67	.82 [.74, .91]	.18
Strangers I1	1.68	0.96	.60	.68 [.58, .79]	.32
Generic I2	1.85	1.01	—	1	
Family I2	1.43	0.78	.52	.64 [.51, .76]	.36
Friends I2	1.44	0.78	.56	.62 [.52, .73]	.38
Work I2	1.69	0.96	.70	.89 [.81, .95]	.11
Strangers I2	1.71	1.00	.61	.71 [.61, .82]	.29
Generic I3	2.91	1.17	—	1	
Family I3	2.41	1.24	.53	.67 [.57, .76]	.33
Friends I3	2.12	1.13	.56	.81 [.72, .90]	.19
Work I3	2.30	1.15	.58	.78 [.69, .88]	.22
Strangers I3	2.35	1.20	.55	.72 [.62, .81]	.28
Generic I4	2.15	0.99	—	1	
Family I4	1.82	0.98	.49	.64 [.54, .75]	.36
Friends I4	1.74	0.90	.58	.82 [.74, .91]	.18
Work I4	2.09	1.07	.70	.83 [.75, .90]	.17
Strangers I4	2.02	1.08	.67	.84 [.75, .92]	.16
Generic I5	1.45	0.68	—	1	
Family I5	1.46	0.71	.48	.78 [.68, .87]	.22
Friends I5	1.42	0.66	.53	.87 [.77, .94]	.13
Work I5	1.71	0.87	.52	.91 [.81, .97]	.09
Strangers I5	1.80	0.96	.50	.79 [.68, .89]	.21

*Note.* I = item; Manifest Correlation = manifest correlation with corresponding generic item; CI = confidence interval based on 1,000 bootstrap draws.

On average, the generic Machiavellianism indicators shared 60.0% (family; CI = [55.0, 64.9]), 69.4% (friends; CI = [65.0, 74.1]), 80.8% (work; CI = [77.1, 84.3]), and 69.2% (strangers; CI = [64.4, 74.1]) true-score variance with their contextualized versions. Thus, Machiavellianism items tended toward a stronger hidden work framing as opposed to other framings. However, consistency patterns varied across the Machiavellianism items (see Table 2 and Figure 3). For example, item 3 (“It’s wise to keep track of information that you can use against people later”, Jones & Paulhus, 2014, p. 38) showed high consistency with the work context (.72), but a considerably lower consistency with the family context (.36). Consistency was more homogeneous for generic item 5 (“Most people can be manipulated”, Jones & Paulhus, 2014, p. 38), which ranged from .90 (family) to .98 (friends).

The generic narcissism indicators shared 74.3% (family; CI = [68.5, 79.1]), 73.6% (strangers; CI = [67.3, 79.3]), 85.2% (friends; CI = [80.9, 89.5)], and 85.3% (work; CI = [80.5, 90.5]) true-score variance with the contextualized versions, on average. Hence, narcissism items tended toward a stronger friend- and work-related than a family- and strangers-related hidden framing. Again, the consistency patterns varied across indicators (see Table 3 and Figure 4). For instance, the consistency pattern for item 2 (“I know that I am special because everyone keeps telling me so”, Jones & Paulhus, 2014, p. 38) was more discrepant, ranging from .47 (family) to .73 (work), than the pattern for item 5 (“I insist on getting the respect I deserve”, Jones & Paulhus, 2014, p. 38), which ranged from .92 (strangers) to .98 (friends).

On average, the generic psychopathy items shared 63.9% (family; CI = [58.1, 69.0]), 72.5% (friends; CI = [.66.7, 77.5]), 84.5% (work; CI = [80.7, 88.0]), and 74.6% (strangers; CI = [69.4, -79.2]) true-score variance with the contextualized versions. The consistency patterns again varied across indicators (see Table 4 and Figure 5). For example, the pattern for item 2 (“Payback needs to be quick and nasty”, Jones & Paulhus, 2014, p. 38) ranged from .62 (friends) to .89 (work) and was less homogeneous than for item 5 (“I’ll say anything to get what I want”, Jones & Paulhus, 2014, p. 38), which ranged from .78 (family) to .91 (work).

Taken together, there was considerable item heterogeneity in the pattern of consistency coefficients. For some DT items, the explained variance in the contextualized versions differed as much as 36% between contexts (Machiavellianism item 3). For other items, the largest difference in the explained variance was only 6% (narcissism item 5). This suggests that the generic DT items contain differing degrees of hidden framings.

#### RQ2—Consistency Across DT Traits

RQ2 focused on comparing the consistency coefficients *across the DT traits* (as opposed to the within-trait perspective addressed in RQ1). The findings indicate that Machiavellianism and psychopathy showed similar patterns of consistency coefficients. On average, generic Machiavellianism and generic psychopathy indicators shared considerably more true-score variance with the work-framed items (80.8%; CI = [77.1, 84.3] and 84.5%; CI = [80.7, 88.0], respectively) than with the family-framed items (60.0%; CI = [55.0, 64.9] and 63.9%; CI = [58.1, 69.0], respectively). Conversely, narcissism showed a more homogeneous pattern of explained variance across contexts, on average (varying from an average of 74.3–85.3% across contexts). The results suggest that generic narcissism shares a rather homogeneous amount of variance with various contexts, while generic Machiavellianism and psychopathy appear to represent different contexts differently.

#### RQ3—Correlation of Framing-Specific Method Factors

RQ3 addressed how framing-specific method factors are intercorrelated within and across DT traits. Table 5 highlights all intercorrelations of the latent variables as estimated by the CT-C(M − 1) model (as derived from Model 2d). We highlight the most important coefficients using differently shaped boxes.

**Table 5.** Variances (Diagonal) and Correlations of the Latent Variables Obtained From Final Model 2d (see Figure 5).

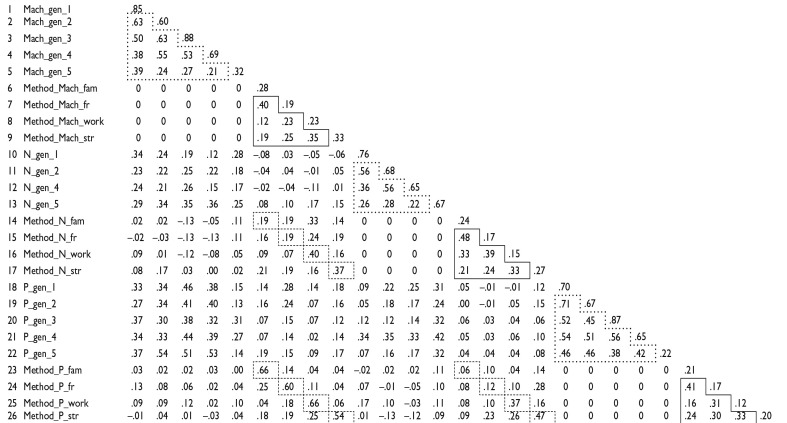

*Note.* Dotted line boxes highlight the intercorrelations of the generic reference items within each DT trait. Dashed line boxes highlight method factor correlations focusing on the same context *across* the DT traits. Solid line boxes highlight method factor correlations within each DT trait. DT = Dark Triad; Mach = Machiavellianism; N = Narcissism; P = Psychopathy; gen = generic framing; fam = family contextualization; fr = friends contextualization; work = work contextualization; str = strangers contextualization.

Solid line boxes highlight method factor correlations within each DT trait. These correlations indicate how far context effects generalize *within* a particular DT trait. Method factor correlations for Machiavellianism varied from .12 to .40, for narcissism from .21 to .48, and for psychopathy from .16 to .41. These results speak for low to moderate generalizability of context effects within a DT trait.

Dashed line boxes highlight method factor correlations focusing on the same context *across* the DT traits. Family method factor correlations ranged from .06 to .66. Friends method factors correlated from .12 to .60. Work method factors correlated from .37 to .66, and strangers method factors from .37 to .54. Of note, the method factors belonging to Machiavellianism and psychopathy that focused on the same context correlated considerably (*r* = .54 to .66). In contrast, method factors belonging to the same contexts for Machiavellianism and psychopathy shared less variance with the corresponding narcissism method factors (.06 to .47). So, there was differential generalizability of context effects across the DT traits: the effects were more common among Machiavellianism and psychopathy.

Dotted line boxes highlight the intercorrelations of the generic reference items within each DT trait. These correlations can be interpreted as measures of convergent validity of the generic DT items. The correlations of the generic Machiavellianism items ranged from .21 to .63. Correlations between the generic narcissism indicators ranged from .22 to .56. Finally, correlations between generic psychopathy items ranged from .38 to .71. So, there was substantial heterogeneity in the convergent validities of generic items within each DT trait.

#### Ancillary Analyses

As many DT studies rely on manifest scale scores, we computed the mean across items with the same framing (e.g., mean of all generic psychopathy items, mean of all psychopathy items with the same context framing). We correlated the generic and contextualized scale scores and investigated if hidden framings persist in the generic DT facets. Details regarding this analysis can be found in the supplements (Sections 5–7). As a high-level summary, the analyses mirrored the findings of the item level: hidden framings were still detectable when correlating generic and contextualized scale scores. Regarding Machiavellianism and psychopathy, the family-framed scale score showed the lowest and the work-framed scale score the largest correlation with the generic version.

Second, we investigated if hidden framings in the DT scales are consequential for the DTs predictive power and used interpersonal deviance (ID) as a criterion. For each DT construct, we estimated eight linear regression models. The first four regressions used a generic DT scale score as a predictor variable (e.g., generic psychopathy) and each of the four ID scales as a criterion (framed as family, friends, work, or strangers). The second set of four regressions exchanged the generic DT scale score with the DT scale score that matches the context of the ID criterion (e.g., family-framed narcissism as a predictor of family-framed ID). An adjusted alpha of 0.05/8 = .00625 (eight tests per trait) accounted for multiple testing. We compared the *R*^2^ from these models descriptively.

Table 6 summarizes the results of the linear regression models. The generic DT score was significantly associated with all but one contextualized ID score (the exception being generic narcissism being a nonsignificant predictor of family-framed ID). However, there were differences in the proportion of explained variance depending on the criterion. On average, the generic DTs explained the lowest variance in family-framed ID (mean *R*^2^ = 4.6%). The explained variance was larger for friends and strangers ID (mean *R*^2^ = 8.9% and 9.5%, respectively) and largest for work-framed ID (mean *R*^2^ = 10.5%), on average. Given this result, a researcher could assume that the DT may be less relevant for predicting family-framed ID than the other three contextualized ID criteria. However, prior analyses identified hidden framings in the generic DT items and aggregates: Family-contextualized DT showed the lowest correlation with the generic DT, which might be one reason for the lower predictability of family-framed ID by the generic DT (= larger contextual asymmetry).

**Table 6.** Standardized Regression Weights and Explained Variance in Interpersonal Deviance Criteria by the Generic and Contextualized Dark Triad (Path Models, Simple Linear Regression).

Predictor	Criterion							
	ID family		ID friends		ID work		ID strangers	
	Beta	*R*^2^ (%)	Beta	*R*^2^ (%)	Beta	*R*^2^ (%)	Beta	*R*^2^ (%)
Mach								
Generic	**0.19**	3.6	**0.28**	7.6	**0.31**	9.4	**0.31**	9.8
Context-congruent	**0.34**	11.5	**0.37**	13.9	**0.44**	19.2	**0.39**	15.0
*R*^2^ (%)—Difference		+7.9		+6.3		+9.8		+5.2
Narc								
Generic	0.07	0.5	**0.14**	1.9	**0.18**	3.2	**0.15**	2.4
Context-congruent	**0.14**	1.9	**0.14**	2.0	**0.25**	6.5	**0.18**	3.3
*R*^2^ (%)—Difference		+1.4		+0.1		+3.3		+0.9
Psych								
Generic	**0.31**	9.6	**0.42**	17.3	**0.43**	18.8	**0.40**	16.4
Context-congruent	**0.48**	22.9	**0.49**	23.7	**0.56**	31.0	**0.49**	24.1
*R*^2^ (%)—Difference		+13.3		+6.4		+12.2		+7.7

*Note.* Bold standardized regression weights were significant (*p* < .00625). Reliability estimates (and confidence intervals [CI] for the Dark Triad are: Mach: gen ω = .68 CI = [.65, .72], family ω = .73 CI = [.70-, 76], friends ω = .74 CI = [.71, .77], work ω = .79 CI = [.76, .81], strangers ω = .77 CI = [.75, .79]; Narc: gen ω = .53 CI = [.48, .58], family ω = .59 CI = [.55, .63], friends ω = .58 CI = [.54, .62], work ω = .60 CI = [.56, .64], strangers ω = .59 CI = [.54, .62]; Psych: gen ω = .71 CI = [.68, .74], family ω = .76 CI = [.73, .79], friends ω = .77 CI = [.73, .80], work ω = .80 CI = [.78, .83], strangers ω = .81 CI = [.78, .83]. Reliability estimates for ID are: family ω = .80 CI = [.77, .83], friends ω = .78 CI = [.75, .82]), work ω = .80 CI = [.76, .83], strangers ω = .81 CI = [.78, .83]. DT= Dark Triad; Mach = Machiavellianism; Narc = Narcissism; Psych = Psychopathy; ID = Interpersonal Deviance; ω = reliability coefficient omega (see Dunn et al., 2014; [] brackets show confidence intervals based on 1,000 bootstrap draws), Beta = standardized regression weight; *R*^2^ (%) = percentage of explained variance in the criterion variable; *R*^2^ (%)—Difference = difference in *R*^2^ between the model including only the generic DT trait and only the context-congruent DT trait as predictor; CI = confidence interval.

Framing the DT scales to the context of the criterion measure increased the explained variance, on average (as indicated by the *R*^2^ differences with an average of +6.2% increase in *R*^2^ across all DTs and IDs; see Table 6). Notably, the context-matching contributed to a more balanced *R*^2^ across some contexts. For instance, context-congruent psychopathy explained almost the same amount of variance in ID family (22.9%), friends (23.7%), and strangers (24.1%). Based on this result, a researcher would likely *not* conclude that psychopathy is less relevant as a predictor of family-framed ID than the other two criteria. This result highlights that it is important to keep potential hidden contextual asymmetries in mind when relating generic predictors and contextualized criteria.

## General Discussion

Many DT items in self-report inventories focus on interpersonal interactions (e.g., SD3, Jones & Paulhus, 2014). Typically, a generic item wording is used, framing participants to consider their DT-related behavior toward, for instance, “others” or “people” in general. Thus far, it remained unclear if respondents mentally average their behavior across interaction partners with equal weight or if their judgments contain interaction partner-specific tendencies. In the current study, we sought to identify such “hidden” interpersonal framings in generic DT items as recently proposed by Schulze et al. (2021). Using generic and contextualized items from the SD3 inventory, we found that generic ratings differed in consistency across contexts. Furthermore, aggregated DT scores related differentially to interpersonal deviance. Our findings highlight that hidden framings exist in some generic DT items and can potentially be consequential for the predictive power of generic DT traits.

### Theoretical Contributions

The current study advances our theoretical understanding of generic and contextualized DT measurements. We found mixed support for the assumption that generic SD3 indicators contain a hidden interpersonal framing (RQ1). For example, the third generic Machiavellianism item as assessed in our study (“It’s wise to keep track of information that you can use against people later”, Jones & Paulhus, 2014, p. 38) could explain 36% of the true-score variance in the family-contextualized version but 72% of the true-score variance in the work contextualized version. This finding speaks for context-specificity in the generic ratings. In contrast, other DT items showed a homogeneous pattern of consistency coefficients (e.g., “I insist on getting the respect I deserve”, Jones & Paulhus, 2014, p. 38), which means that the generic item predicted all contextualized versions almost equally well. This pattern either speaks for a more balanced mental aggregation of contexts or implies high cross-contextual consistency of the DT aspect. Notably, if the respondent’s behavior does not vary much as a function of the context, the hidden contextual framing may not matter much. Importantly, items of the same DT facet did not necessarily show homogeneous patterns of consistency. Thus, different items of a DT facet may be differentially affected by contextual framings. In conclusion, our results suggest that individuals may not always give equal weight to different interaction partners while rating their own DT behavior, supporting the assumptions made by Schulze et al. (2021). Rather, based on the item under consideration, the generic rating represents some interaction contexts better than others. Thus, our analysis helps to shed some light on the “black box” of generic DT ratings and calls for further research into the mental response processes (see also Angleitner et al., 1986).

The differences in consistency patterns among DT items potentially carry importance for scale construction. In the item construction process, researchers put much weight on considering the universe of behaviors, thoughts, and feelings associated with a DT construct (e.g., manipulating, getting revenge; Jones & Paulhus, 2014). However, the universe of contexts relevant to the behavior is usually not as elaborately specified (De Raad, 2005; Rauthmann, 2015). For items that show a homogeneous consistency pattern, a generic definition of the relevant context, such as “others” or people,” may suffice as the generic item captures behavior in different contexts equally well. However, with a heterogenous consistency pattern, predictions and interpretation become more complicated as the generic scores are differentially context-laden. One approach—the one we took in the current study—is to explore hidden framings in the generic items. By exploring the context-ladenness of generic DT items, we can provide clearer interpretations concerning the relationship between items and scale scores with external criteria. Another approach would be to construct items that draw on the universe of contexts that are relevant to the behavioral expression of the DT. This would allow to build items that are less abstract regarding context, reducing the possibility for idiosyncratic interpretations of respondents. As a potential downside of this approach, not all items might apply to all individuals based on their particular life circumstances (Schulze et al., 2021). Future research must identify the optimal level of abstraction and concreteness in the item formulation of the DT.

Our study also revealed low to moderate convergence between the generic reference items, suggesting that the indicators within *the same* facet assess rather different aspects. For instance, although the items “It’s wise to keep track of information that you can use against people later” and “Most people can be manipulated” are both considered indicators of Machiavellianism (see Jones & Paulhus, 2014, p. 38), their consistency patterns differed. For homogeneous indicators, we would expect high intercorrelations and similar consistency patterns. In this sense, our analyses show that investigations on the DT item level may reveal important new insights that remain undetected when only focusing on what the different DT indicators have in common. Thus, the item-level analysis provides an additional, so far understudied perspective on the DT that complements perspectives on the broader traits.

Similar to the generic items, interpersonal contexts in which the traits are expressed also seem not interchangeable. The method factor correlations from the latent variable models differed, suggesting that the contexts are structurally different (RQ3). Latent variable models such as the CT-C(M − 1) model allow to separate context variance with a psychometric sound meaning and thus complement available methods which can be employed to assess the functionality of items of the DT across contexts (Eid et al., 2016).

Generic DT items were better able to explain true-score variance in work-contextualized indicators than in the other three interpersonal framings, on average. This was particularly true for Machiavellianism and psychopathy indicators but less for narcissism indicators (RQ2). In addition, method factors for Machiavellianism and psychopathy correlated more strongly with each other than with the method factors of narcissism. These findings align with past research that emphasized the conceptual closeness of Machiavellianism and psychopathy on the one hand and the distinctiveness of these two facets from narcissism on the other (Vize et al., 2018). Another explanation for this pattern of findings might be that the SD3 (and, as a consequence, our subset of SD3 items) does not discriminate adequately between Machiavellianism and psychopathy (e.g., Sharpe et al., 2021). We further discuss this possibility in the limitations section.

The diverging correlations between generic and contextualized ratings in the latent variable models are also reflected in the diverging correlations among generic and contextualized mean scale scores, particularly for Machiavellianism and psychopathy. Concerning these traits, the generic scale score showed the lowest correlation with a family-context scale score and the highest correlation with a work-context scale score (see supplements for details). In line with assumptions made by Schulze et al. (2021), item aggregation does not necessarily balance out hidden framings completely. Rather, aggregation of generic DT indicators containing different hidden framings may cancel out some contextual variability more readily (i.e., family-related variability) than other sources of variability (i.e., work-related variability). This cancelation remains hidden if researchers focus on aggregation principles *after* the mental aggregation processes conducted by respondents (see also Uher, 2018, for noting that the mental process behind choosing a personality item response is not well understood).

Hidden framings in aggregated scores may potentially have practical consequences for our interpretation of the predictive power of generic DT traits. Our exploratory regression analyses revealed that the predictive power of the DT increases if the criterion and predictor comprise the same contextual framing. In line with prior frame-of-reference research in organizational psychology (e.g., Lievens et al., 2008), this finding suggests that in some cases, predictive power could potentially be increased by framing the DT ratings to the context of the criterion. This effect may be more pronounced when the original generic items contain hidden contextual framings that are *asymmetric* to the contextual framing of the criterion. However, our findings go beyond providing support for the frame-of-reference effect: Recall that for Machiavellianism and psychopathy, both at the item and scale level, the correlation between family-framed scores and generic scores was the lowest relative to the other contexts on average. As a consequence, a researcher unaware of this finding may potentially underestimate the capability of the constructs to predict criteria from the family-context relative to criteria from other contexts (e.g., work). The prediction of other clinically relevant criteria in the DT domain may involve similar mechanisms.

### Future Research

More research is needed to identify the contextual features contributing to hidden framings. We hypothesized that test-takers associate certain DT-related behavior, feelings, or beliefs with certain situations and that some situations/contexts are weighted more heavily by individuals during the response process. These assumptions need further investigation. In their prototype analysis of situations, Cantor et al. (1982) made the important observation that individuals have prototypical features in mind when asked to imagine a certain kind of situation (e.g., a party situation or a religious ceremony). Specifically, Cantor et al. asked individuals to imagine a situation for a given stimulus phrase and write down features of these situations that came to their minds (imagery task). This methodology could be adapted to identify dominant or prototypical contexts individuals associate with certain DT aspects. For instance, researchers could ask participants to write down the first situation that comes to their mind in which a specific DT behavior is relevant (see also De Raad, 2005). Descriptions of such prototypical situations may help explain why generic scores better represent certain situations (e.g., work) than others.

Similarly, probing methodology may help understand which contexts individuals consider when they rate DT items (see Jobe, 2003 for an overview of cognitive interviewing techniques). The finding that respondents considered certain interpersonal contexts more often than others (e.g., work more often than family) when completing the item rating would provide some tentative evidence that prototypical contexts contribute to the emergence of hidden framings. Ambulatory assessment methodology (Trull & Ebner-Priemer, 2014) could also be used to identify the situational triggers of DT behavior and their frequency in different interpersonal situations (e.g., as in Nübold et al., 2022). Nübold et al. (2017) published an interesting study protocol on developing a taxonomy comprising situational triggers for the expression of the DT at work, but to our knowledge, this taxonomy still awaits realization.

Taking a broader perspective beyond the DT, we agree with Murtha et al. (1996, p. 205) who stated that “it is important to aggregate the correct responses (i.e., content) across the appropriate situations such that the aggregated items share variance from the same latent situational and content factors as the criteria.” As generic personality items are oftentimes unspecific regarding the contexts to consider in the response process, we usually do not know how much and what kind of situational variance these measures capture (see also Schulze et al., 2021). In this regard, our study sheds some light on the situational variance captured by a selected set of DT items. We advocate for more research in personality psychology to improve our understanding of context-ladenness and the predictive power of generic trait measures (Schulze et al., 2021). For instance, items assessing facets of extraversion and agreeableness include many items with interpersonal situations as reference (see also Saucier & Conley, 2015). The methods of the current study may be applied to identify hidden framings in other personality traits. Further research on the contexts relevant to personality expression (e.g., Saucier et al., 2007) would be helpful to construct appropriate context tags for other personality items that do not focus on interpersonal situations.

An anonymous reviewer noted that literature critically discussed the term “Dark Triad.” For instance, Rose et al. (2023) took the view that the Dark in Dark Triad “is sensationalistic, stigmatizing, and provides little to no guidance as to what constructs do and do not fall under its purview” (p. 782). They suggested the “Antagonistic Triad” as a less stigmatizing alternative highlighting antagonism as the common part of the three constructs. We want to add to this perspective that the term “Dark” ignores the potentially positive sides of specific facets. For instance, narcissism in the context of leadership comes with positive as well as negative effects (Fatfouta, 2019). Although we support establishing a less stigmatizing language, we decided to discuss this matter but stick to the term “Dark Triad” throughout the manuscript for two reasons: Foremost, our primary measurement instrument, the Short Dark Triad, carries the term in the title. We aligned our wording to this measure to avoid confusion. Second, the term is well-established in the literature and simplifies communication among researchers. We encourage using the most recent measures such as the Five-Factor Model Antagonistic Triad Measure (Rose et al., 2023) that not only do better in discriminating the facets but also may help establish a less stigmatizing language over time.

### Limitations

Our study has several limitations. The *first* limitation concerns the generalizability of our findings because of the employed measurement. We only investigated context effects using a specific subset of one specific questionnaire. The current approach was selected because (a) the SD3 is one of the most frequently used instruments in the DT literature and provides a compromise between length and completeness (reducing respondent burden was a major aim of chosing the SD3; more items may have resulted in other problems such as more pronounced careless responding or survey fatigue), and (b) we were mostly interested in item-specific effects rather than the full latent traits. Thus, whether our findings generalize to the complete SD3 and other DT measurement instruments remains unknown. Moreover, studies have criticized the SD3 for providing only a limited view of the DT and not assessing Machiavellianism and psychopathy in a distinct way (see e.g., DeShong et al., 2017; Miller et al., 2017; Rose et al., 2023; Sharpe et al., 2021). As stated by Miller et al. (2017, p. 450), “existing measures of Machiavellianism are actually measuring psychopathy” (for exceptions see the instruments by Rose et al., 2023; Sharpe et al., 2021). Thus, similar patterns of consistency coefficients between psychopathy and Machiavellianism might be related to measurement issues inherent in the SD3. Indeed, we also found large correlations between Machiavellianism and psychopathy items and method factors, supporting this view. At the same time, the Machiavellianism and psychopathy method-factor correlations for the same contexts, although high, were far from unity. This finding indicates that method effects did generalize completely across both traits, which needs further investigation in future research.

Although the SD3 has conceptual issues and our item pool was small, we included prototypical items to measure the DT. For instance, the Narcissistic Personality Inventory 16 includes the item “I know that I am good because everybody keeps telling me” (Ames et al., 2006, p. 449), which is similar to our included SD3 indicator. As another example, the Triarchic Psychopathy Measure (Patrick, 2010) and the MACH-IV (Christie & Geis, 1970) include items that describe influencing or manipulating others. As a last example, the Dirty Dozen assessment (Jonason & Webster, 2010) also contains item wordings similar to those of our SD3 items (but has also measurement problems, see Maples et al., 2014; Miller et al., 2012). Recognizing this similarity, we hypothesize that hidden framings are important for other DT measures. Based on our arguments, we advocate for more research investigating the relevance of hidden framings in other DT assessments, especially in instruments that provide better discrimination between the three DT traits (Rose et al., 2023; Sharpe et al., 2021).

The *second* limitation concerns the generalizability of our findings because of the employed sample. We investigated a sample from the general population where most participants scored low to moderate on the trait measures. This questions the generalizability to individuals showing higher DT scores. Although we intended to study the hidden framing phenomenon in a sample derived from the general population, this is an important limitation that needs to be addressed in future research. We hypothesize that item-level consistency coefficients may be more balanced for individuals who score high on the DT traits. An individual scoring high on psychopathy measurements, for example, may agree strongly to an item such as “Payback needs to be quick and nasty” (Jones & Paulhus, 2014, p. 38) independently of the context or situation, because those individuals likely express DT behaviors consistently in a variety of contexts. The generalizability of our findings may also be limited regarding other participant characteristics besides their trait standing. Specifically, we employed a noncommercial German online panel for psychological studies (test-takers had agreed to participate voluntarily in psychological research). Thus, it is likely that only highly motivated individuals responded to our survey questions (as also indicated by the rather low proportion of careless responses). Despite these limitations, the variance in gender and age in our study speaks for a certain degree of diversity and representativeness. However, we encourage further studies to investigate the generalizability of our findings in other samples (e.g., samples with clinical or forensic backgrounds or samples collected using traditional sampling procedures not based on online questionnaires).

The *third* limitation concerns the context tags we employed. Although context tags contribute to a higher standardization of item interpretation across individuals (Lievens et al., 2008), they still allow for interpretational differences (e.g., Schulze et al., 2021). In our specific case, using the item tag “persons you work with,” may refer to, for example, supervisors, clients, and coworkers. It remains unknown if our results generalize to these subgroups of interaction partners. In future studies, more specific contextual framings could be used and contrasted against the broader ones. Such analyses may help to identify the optimal level of contextualization that represents the best trade-off between specificity and generality.

*Fourth*, social desirability may be another factor that contributes to a differential correlation pattern of the generic scores with the contextualized versions. Considering that “faking occurs due to an interaction between person and situation” (Ziegler & Buehner, 2009, p. 548), contextualized DT items could be differentially prone to socially desirable responding. For example, individuals may shy away from responding honestly to questions that ask if they manipulate their family members or take revenge on them because of the strong social stigma associated with such behavior. This stigma could be less pronounced in the working context, resulting in a context-specific social desirability effect. Future research should examine if contextualized DT items are differentially prone to socially desirable responses and if such differences explain variability in consistencies.

*Fifth*, although the use of contextualized personality inventories is standard practice in personality research (e.g., Grover & Furnham, 2021; Lievens et al., 2008), the method comes with the disadvantage that item redundancy may influence the response process during the survey administration (Baird & Lucas, 2011). Our study presented the questionnaires in randomized block order to alleviate this problem. In future research, the questionnaires could be given at several different time points to reduce these effects further.

*Sixth*, the CT-C(M − 1) model with indicator-specific latent variables, like all multimethod models (see Eid et al., 2003, 2008 for reviews), comes with assumptions that may be violated in practice. For example, the CT-C(M − 1) model by Eid et al. (2003) assumes homogeneous method effects. However, our findings indicate that the method effects are not perfectly homogeneous (e.g., the method factor loadings vary across indicators), and thus reliability and method-specificity may be underestimated (Geiser & Simmons, 2021). This may impact our conclusions concerning hidden framings in the current study as those were based on the estimated consistency and specificity coefficients—the latter being subject to potential model bias. Therefore, further studies should investigate the homogeneity of method effects by assessing individual DT aspects with multiple homogeneous indicators.

### Conclusion

Our study suggests hidden interpersonal framings in generic DT items. Individuals do not seem to aggregate mentally across different interaction partner groups with equal weight. This effect was item-specific and did not generalize to all DT indicators. We call for more research focusing on the DT at the item level *in combination* with the aggregated scale level. As DT indicators of the same trait show only low convergent validity and therefore seem structurally different rather than interchangeable, item-level analyses may complement perspectives on the broader traits. Researchers should be aware that hidden framings can be consequential. In our study, contextual specificity in DT scale scores impacted their predictive power in explaining a frequently measured criterion, interpersonal deviance. Future research needs to test for alternative explanations of our findings and probe the generalizability of the results to other DT items, assessment instruments, and samples.

**Figure 6.**
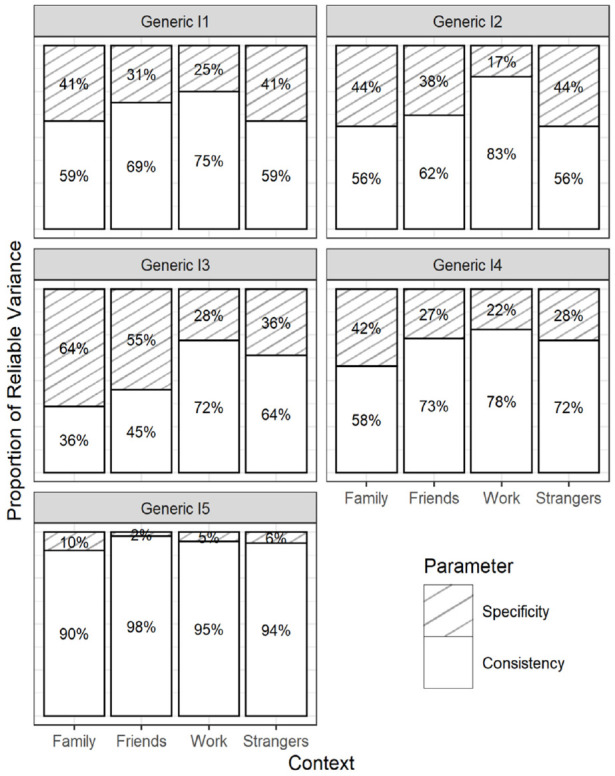
Item-Level Consistency and Specificity Estimates From the Full Correlated Trait-Correlated (Method − 1) Model With Indicator-Specific Latent Variables (Eid et al., 2008) for (Panel A) Machiavellianism; (Panel B) Narcissism; (Panel C) Psychopathy. *Note*. The coefficients add up to 100%. The coefficients can be interpreted as the proportion of true-score variance that generic DT items can explain(consistency) and cannot explain (specificity) in their contextualized versions. DT = Dark Triad.

**Figure 7.**
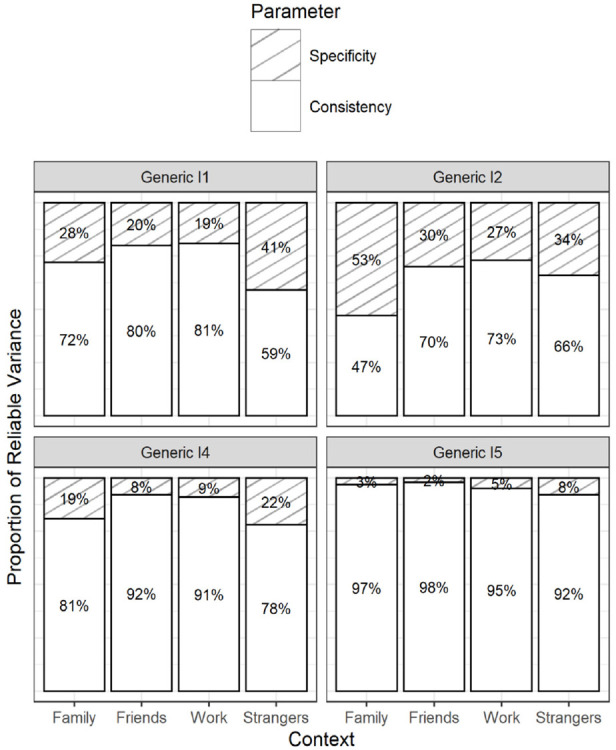
Item-Level Consistency and Specificity Estimates From the Final Latent Variable Model 2d (see Figure 5) − Narcissism. *Note*. The coefficients add up to 100%. The coefficients can be interpreted as the proportion of true-score variance that the generic items can explain (consistency) and cannot explain (specificity) in their contextualized versions. DT = Dark Triad.

**Figure 8.**
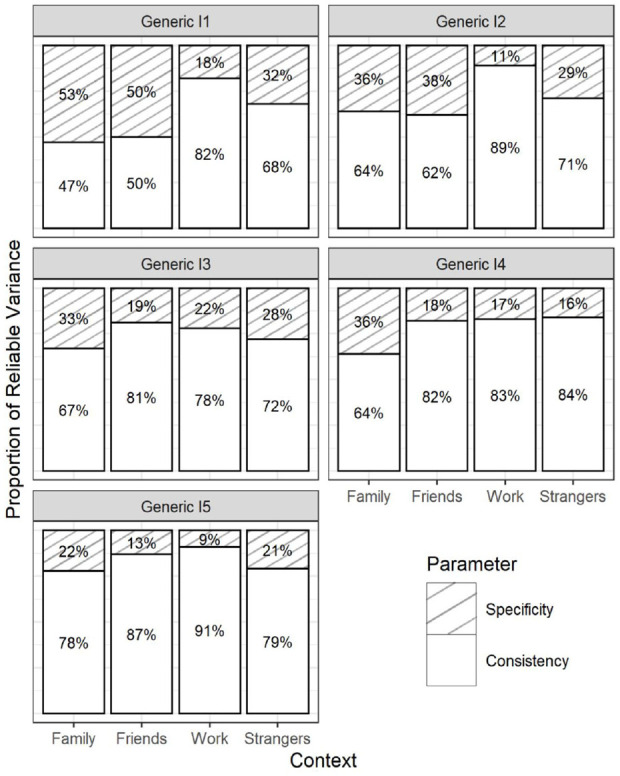
Item-Level Consistency and Specificity Estimates From the Final Latent Variable Model 2d (see Figure 5) − Psychopathy. *Note*. The coefficients add up to 100%. The coefficients can be interpreted as the proportion of true-score variance that the generic items can explain (consistency) and cannot explain (specificity) in their contextualized versions. DT = Dark Triad.

## Supplemental Material

sj-docx-1-asm-10.1177_10731911231220357 – Supplemental material for Uncovering Hidden Framings in Dark Triad Self-Ratings: What Frames-of-Reference Do People Use When Responding to Generic Dark Triad Items? Supplemental material, sj-docx-1-asm-10.1177_10731911231220357 for Uncovering Hidden Framings in Dark Triad Self-Ratings: What Frames-of-Reference Do People Use When Responding to Generic Dark Triad Items? by Julian Schulze, Manuel Heinrich, Jan-Philipp Freudenstein, Philipp Schäpers and Stefan Krumm in Assessment

## Notes

1. We acknowledge that dark triad aspects can be displayed in noninteractional contexts, too (e.g., staring in the mirror as an indicator for narcissism; cheating on tax returns as an indicator for Machiavellianism; Jones & Paulhus, 2011). These indicators are not in the focus of the current study.

2. Our preregistered research questions use terminology from the correlated trait-correlated (method − 1) model (CT-C[M − 1]) with indicator-specific latent variables (see Eid et al., 2008). This model decomposes variance of the contextualized items into (a) reliable variance shared with the generic framing (consistency) and (b) reliable variance not shared with the generically framed items (specificity, Eid et al., 2008). The CT-C(M − 1) model considers potential item wording effects (see, e.g., Xu et al., 2013 for reasons to consider parallel wording).

3. A sensitivity analysis was conducted including those individuals with more than two failed attention checks and with individuals with partial data on the contextualized questionnaires. The results did not materially differ from the primary results as reported in the main paper (see supplemental material for details).

4. As pointed out in the preregistration, the scale anchors for ID items were adapted to a 6-point scale (1 = *never*—6 = *very frequently*; a similar approach has been taken by Sackett et al., 2006, who also used a—4-point—Likert-type scale). The scale anchors for the DT items were in line with Malesza et al. (2019), who used a 5-point Likert-type scale (1 = *strongly disagree*—5 = *strongly agree*).
