# Supplementary material for: Uncovering Hidden Framings in Dark Triad Self-Ratings: What Frames-of-Reference Do People Use When Responding to Generic Dark Triad Items?
Source: Assessment. 2024 Jan 29;31(7):1472–92. doi: 10.1177/10731911231220357 (PMC11411850; doi:10.1177/10731911231220357)
Supplement: sj-docx-1-asm-10.1177_10731911231220357 – Supplemental material for Uncovering Hidden Framings in Dark Triad Self-Ratings: What Frames-of-Reference Do People Use When Responding to Generic Dark Triad Items? [file sj-docx-1-asm-10.1177_10731911231220357.docx]

**Supplemental Material for the Article**

**Uncovering Hidden Framings in Dark Triad Self-Ratings: What Frames-of-Reference do People use When Responding to Generic Dark Triad Items?**

**Table of Contents**

[1) Pilot Study 3](#_Toc139192185)

[2) Pilot Study – Measurement Invariance Tests 7](#_Toc139192186)

[3) Main Study – Convergent Validity of Narcissism Item 3 8](#_Toc139192187)

[4) Main Study – Sensitivity Analyses 9](#_Toc139192188)

[5) Ancillary Analyses – Methodology and Results 10](#_Toc139192189)

[6) Ancillary Analyses – Descriptives, Reliability Estimates, and Intercorrelations 11](#_Toc139192190)

[7) Ancillary Analyses – Confidence Intervals (Correlation Differences) 12](#_Toc139192191)

[References 13](#_Toc139192192)

# Pilot Study

The pilot study served two purposes: 1) To create context tags covering a reasonable spectrum of everyday interaction partners that could be used to contextualize DT items. 2) To identify a manageable set of generic DT items suitable for contextualization and for use in our latent variable models of the main study. Code to replicate the analyses can be downloaded from the [OSF project](https://osf.io/3rz8m/?view_only=c6b61933ca5b46929c903d0845a3bd40).

***Context-Tag Creation***

First, it was necessary to develop context tags capturing different groups of interaction partners as most Short Dark Triad items (SD3; the primary measurement instrument that we used in our study) refer to interpersonal situations.

We constructed four tags, each relating to the situational domain of interpersonal associations as identified by Saucier et al. (2007): (1) Persons from the family (hereinafter referred to as "family"), (2) persons from the circle of friends ("friends"), (3) persons you work with ("work"), and (4) persons you hardly know ("strangers"). These four interpersonal contexts were chosen due to their relevance for the DT, their broad applicability, and in order to cover a reasonable spectrum of everyday interaction partners (see, e.g., Wrzus et al., 2016 for an experience-sampling study capturing the frequency of interactions with these groups).

***Construction of a Suitable Item Pool***

**Method**

***Sample***

We used a publicly available dataset of a study that used an English version of the SD3 questionnaire as a measurement instrument (https://osf.io/xey8h/; Vize et al., 2020). Note that although the pilot data were based on the English SD3 version, the main study, conducted in Germany, used the German version of the SD3 (see Malesza et al., 2019). At the time of data collection, we could not identify open data based on the German SD3 version comparable to the Vize et al. (2020) data.

The Vize et al. dataset consists of two subsamples (*n_1_* = 627; *n_2_* = 628). Vize et al. (2020) collected the sample via Amazon Mechanical Turk. The mean age of the full sample was 38.9 years, with a standard deviation of 11.9. The proportion of male participants was 41%.^[[1]](#footnote-1)^

***Procedure***

First, the authors of this manuscript reviewed which SD3 items can be appropriately contextualized to all four interpersonal contexts (using the context tags described above). After multiple discussion rounds, four researchers completed the final round of item reviews. Only SD3 items rated as appropriate for contextualization for all contexts by at least three of four researchers were included in the next step.

Afterwards, we aimed to develop an appropriate latent variable measurement model for an SD3 item pool that is no longer than 15 items to reduce the response burden (five items per DT construct). Above mentioned publicly available samples served for this purpose. Data of subsample 1 was used to reduce the initial item pool based on an item selection procedure (Schultze, 2018). Subsample 2 served for cross-validating the optimal solution. Models with a CFI ≥ .90, RMSEA ≤ .08, and SRMR ≤ .10 were considered acceptable (for an overview, see Schweizer, 2010). We used the maximum likelihood robust estimator to account for skewed data (MLR; West et al., 1995). The R package "lavaan" (Rosseel, 2012; version 0.6-10) was used to fit all latent variable models.

**Results**

Overall, 21 of the initial 27 SD3 items were considered for further examination by the experts (six assessing psychopathy, nine assessing Machiavellianism, and six items assessing narcissism). For example, the items "I enjoy having sex with people I hardly know" and "I have never gotten into trouble with the law" (see Jones & Paulhus, 2014, p. 38) were excluded. The first item already references a specific interaction partner group ("people I hardly know"), and some contextualizations ("family") may have impacted the response process negatively. The second item was excluded because it did not focus on interpersonal situations, specifically. In contrast, items such as " People see me as a natural leader" or "It’s true that I can be mean to others" (see Jones & Paulhus, 2014, p. 38) were considered appropriate for contextualization.

Our analyses using the public datasets revealed that neither a three-factor model including all 27 SD3^[[2]](#footnote-2)^ items nor including those items suitable for contextualization fitted the data well in subsample 1 (robust CFI: .803; robust RMSEA: .081; SRMR: .074 and robust CFI: .806; robust RMSEA: .095; SRMR: .078, respectively). The "bruteforce" function as implemented in the R statistics package "stuart" (Schultze, 2018), was used to prune the item pool to the intended five items per DT construct. Brute-force guarantees to find the best measurement model (following a predefined fit function) for a given data structure and item set (Schultze, 2018). The model fit of this 15-item version was acceptable in subsample 1 (robust CFI: .923; robust RMSEA: .070; SRMR: .049) and in subsample 2 (robust CFI: .924; robust RMSEA: .067; SRMR: .050). We tested metric and scalar measurement invariance across samples as an additional quality check (using R code by Schroeders & Gnambs, 2018). Non-significant chi-square difference tests supported the assumption of measurement invariance (see Section 2 of this supplementary material file). Finally, we computed reliability estimates for the DT scale scores based on all 27 SD3 items and based on the reduced 15-item set: *Full item pool*: In subsample 1 coefficient alpha for Machiavellianism was α = .84 (subsample 2: α = .83), for narcissism α = .82 (subsample 2: α = .83), and for psychopathy α = .82 (subsample 2: α = .80). *Reduced item pool*: Coefficient alpha of the manifest scale score in subsample 1 for Machiavellianism was α = .79 (subsample 2: α = .80), for narcissism α = .70 (subsample 2: α = .70), and for psychopathy = .82 (subsample 2: α = .80). This reduced item pool was employed in the main study.

# Pilot Study – Measurement Invariance Tests

*Tests for metric and scalar measurement invariance across both subsamples collected by Vize et al. (2020). Both Chi-Squared difference tests were non-significant, supporting the assumption of metric and scalar measurement invariance*

|  | df | AIC | BIC | Chisq. | Chisq. diff | df diff | Pr (>Chisq) |
| --- | --- | --- | --- | --- | --- | --- | --- |
| configural | 174 | 51799 | 52292 | 727.99 |  |  |  |
| metric | 186 | 51784 | 52216 | 737.27 | 8.41 | 12 | .753 |
|  |  |  |  |  |  |  |  |
| metric | 186 | 51784 | 52216 | 737.27 |  |  |  |
| scalar | 198 | 51763 | 52133 | 740.19 | 2.92 | 12 | .996 |

*Note.* df = degrees of freedom

# Main Study – Convergent Validity of Narcissism Item 3

*Correlations of the third narcissism item (in a generic, family, friends, work, and strangers version) with the other narcissism items*

| Item | N_gen_3 | N_fam_3 | N_fr_3 | N_work_3 | N_str_3 |
| --- | --- | --- | --- | --- | --- |
| N_gen_1 | 0.18 | 0.11 | 0.07 | 0.15 | 0.14 |
| N_gen_2 | 0.13 | 0.04 | 0.03 | 0.11 | 0.10 |
| N_gen_3 | 1 | 0.56 | 0.67 | 0.74 | 0.67 |
| N_gen_4 | 0.01 | 0.01 | 0 | 0.03 | 0.02 |
| N_gen_5 | 0.10 | 0.02 | 0.02 | 0.09 | 0.04 |
| N_fam_1 | 0.07 | 0.04 | -0.02 | 0.06 | 0.05 |
| N_fam_2 | -0.01 | 0 | -0.06 | -0.06 | -0.04 |
| N_fam_3 | 0.56 | 1 | 0.66 | 0.59 | 0.51 |
| N_fam_4 | 0.06 | 0.01 | -0.02 | 0.07 | 0.02 |
| N_fam_5 | 0.07 | 0.06 | 0.02 | 0.05 | 0.01 |
| N_fr_1 | 0.15 | 0.10 | 0.05 | 0.11 | 0.09 |
| N_fr_2 | 0.06 | 0.02 | -0.02 | -0.03 | 0.01 |
| N_fr_3 | 0.67 | 0.66 | 1 | 0.68 | 0.62 |
| N_fr_4 | 0.06 | 0.04 | -0.03 | 0.08 | 0.05 |
| N_fr_5 | 0.06 | 0.04 | 0.01 | 0.05 | -0.01 |
| N_work_1 | 0.09 | 0.05 | 0.01 | 0.08 | 0.07 |
| N_work_2 | 0.04 | -0.02 | -0.04 | 0.02 | 0.04 |
| N_work_3 | 0.74 | 0.59 | 0.68 | 1 | 0.67 |
| N_work_4 | 0.08 | 0 | 0 | 0.06 | 0.05 |
| N_work_5 | 0.05 | 0.04 | 0 | 0.09 | 0 |
| N_str_1 | 0.05 | -0.01 | -0.05 | 0.05 | 0.01 |
| N_str_2 | 0.09 | -0.02 | -0.02 | 0.06 | 0.04 |
| N_str_3 | 0.67 | 0.51 | 0.62 | 0.67 | 1 |
| N_str_4 | -0.02 | -0.02 | -0.05 | 0.01 | -0.03 |
| N_str_5 | 0.06 | 0.04 | 0.03 | 0.09 | 0.02 |

*Note.* N = Narcissism; gen = generic framing; fam = family contextualization;

fr = friends contextualization; work = work contextualization; str = strangers contextualization

|  |  | **χ²** | **df** | **χ²- p value** | **CFI** | **RMSEA** | **SRMR** |
| --- | --- | --- | --- | --- | --- | --- | --- |
| Models 1 | Machiavellianism + all context factors | 556.078 | 239 | <.001 | 0.971 | 0.041 | 0.030 |
|  | Narcissism + all context factors | 518.190 | 239 | <.001 | 0.974 | 0.039 | 0.041 |
|  | Psychopathy + all context factors | 537.644 | 239 | <.001 | 0.969 | 0.043 | 0.033 |
| Models 2a | Dark Triad + family context factor* | 467.758 | 214 | <.001 | 0.968 | 0.037 | 0.028 |
|  | Dark Triad + friends context factor* | 400.982 | 214 | <.001 | 0.979 | 0.033 | 0.020 |
|  | Dark Triad + work context factor* | 322.715 | 214 | <.001 | 0.990 | 0.025 | 0.018 |
|  | Dark Triad + strangers context factor* | 416.066 | 214 | <.001 | 0.978 | 0.034 | 0.021 |
| Model 2b | Dark Triad + family and friends context factors* | 1113.652 | 629 | <.001 | 0.969 | 0.031 | 0.039 |
| Model 2c | Dark Triad + family, friends, and work context factors* | 2239.365 | 1231 | <.001 | 0.960 | 0.032 | 0.030 |
| Model 2d | Dark Triad + all context factors* | 3502.911 | 2020 | <.001 | 0.956 | 0.030 | 0.036 |

# Main Study – Sensitivity Analyses

*Model fit statistics after re-estimating the latent variable models with the sensitivity dataset (n = 881)*

*Note*. Solution based on Maximum Likelihood Robust estimator (MLR); χ² = chi-square test statistic; df = degrees of freedom; CFI = Comparative Fit Index; RMSEA = Root Mean Square Error of Approximation; SRMR = Standardized Root Mean Square Residual; * Narcissism indicator 3 excluded.

The maximum absolute difference between the loadings of the completers dataset and the sensitivity dataset as estimated by the final Model 2d was .02. The maximum absolute difference between the latent correlations of the completers dataset and the sensitivity dataset as estimated by the final Model 2d was .05.

# Ancillary Analyses – Methodology and Results

As outlined in the main manuscript, we correlated the generic and contextualized mean scores and compared the correlations against each other. Then, for each generic DT score, six tests for differences between paired correlations using the R package "psych" were conducted (Revelle, 2008, e.g., for Machiavellianism: Does the generic-family correlation differ from the generic-work correlation?). An adjusted alpha of 0.05/6 = .0083 (six tests per trait) accounted for multiple testing (Cabin & Mitchell, 2000). These tests investigate if the correlations between the generic and the contextualized scores differ significantly from each other within a particular DT trait (i.e., hidden contextual framings can be differentiated within a DT trait).

Section 6 of the supplemental materials summarizes descriptive statistics, reliability estimates, and intercorrelations of DT scale scores. The grey cells in Section 6 highlight the correlations between each generic DT scale score and the contextualized versions. Section 7 of the supplemental materials shows the 99.17% CIs around the point estimates for the correlation differences. From the total of 18 tests of correlational differences (six per trait), 15 were significant (*p* < .0083; 5 tests for Machiavellianism, 4 tests for narcissism, and 6 tests for psychopathy). The significant correlation differences were small in magnitude ranging from *r*_diff_ = |.05| to |.19|. The family contextualized versions showed the descriptively lowest (*r* = .55-.65), and the work contextualized version the descriptively largest correlation (*r* = .74-.77) with the generic version.

# Ancillary Analyses – Descriptives, Reliability Estimates, and Intercorrelations

*Descriptive statistics and reliability estimates*

|  |  |  |  |  | **Manifest correlations and reliability** | | | | |
| --- | --- | --- | --- | --- | --- | --- | --- | --- | --- |
|  |  | ***M*** | ***SD*** |  | **Generic** | **Family** | **Friends** | **Work** | **Strangers** |
| **Mach** | **Generic** | 2.71 | 0.66 |  | (.68 [.65-.72]) |  |  |  |  |
|  | **Family** | 2.08 | 0.70 |  | .55 | (.73 [.70-.76]) |  |  |  |
|  | **Friends** | 2.05 | 0.67 |  | .66^a^ | .62 | (.74 [.71-.77]) |  |  |
|  | **Work** | 2.69 | 0.80 |  | .74 | .54 | .66 | (.79 [.76-.81]) |  |
|  | **Strangers** | 2.51 | 0.81 |  | .65^a^ | .52 | .62 | .68 | (.77 [.75-.79]) |
| **Narc** | **Generic** | 2.44 | 0.60 |  | (.53 [.48-.58]) |  |  |  |  |
|  | **Family** | 2.58 | 0.67 |  | .65^c^ | (.59 [.55-.63]) |  |  |  |
|  | **Friends** | 2.50 | 0.65 |  | .74^b^ | .72 | (.58 [.54-.62]) |  |  |
|  | **Work** | 2.53 | 0.66 |  | .76^b^ | .66 | .73 | (.60 [.56-.64]) |  |
|  | **Strangers** | 2.28 | 0.65 |  | .69^c^ | .58 | .67 | .70 | (.59 [.54-.62]) |
| **Psych** | **Generic** | 2.07 | 0.67 |  | (.71 [.68-.74]) |  |  |  |  |
|  | **Family** | 1.72 | 0.66 |  | .60 | (.76 [.73-.79]) |  |  |  |
|  | **Friends** | 1.62 | 0.61 |  | .67 | .65 | (.77 [.73-.80]) |  |  |
|  | **Work** | 1.92 | 0.76 |  | .77 | .59 | .70 | (.80 [.78-.83]) |  |
|  | **Strangers** | 1.91 | 0.78 |  | .72 | .57 | .67 | .74 | (.81 [.78-.83]) |

*Note.* Coefficient omega (see Dunn et al., 2014) is presented in the diagonal; manifest scale correlations refer to the generic Dark Triad traits with their contextualized versions (grey).

Mach = Machiavellianism; Narc = Narcissism; Psych = Psychopathy; correlations indexed with identical letters are not significantly different from each other (within a Dark Triad trait); [] brackets show confidence intervals based on 1,000 bootstrap draws.

# Ancillary Analyses – Confidence Intervals (Correlation Differences)

*Confidence intervals for correlation differences*

| **DT Score** | **Comparison** | **Point estimate [99.17% CI for the correlation difference] (*n* = 814)** |
| --- | --- | --- |
|  |  |  |
| **Mach** | Generic - Family versus Generic - Friends | -0.11 **[-0.1724 – -0.0496]** |
|  | Generic - Family versus Generic - Work | -0.19 **[-0.2555 – -0.1279]** |
|  | Generic - Family versus Generic - Strangers | -0.10 **[-0.1688 – -0.0327]** |
|  | Generic - Friends versus Generic - Work | -0.08 **[-0.1318 – -0.0303]** |
|  | Generic - Friends versus Generic - Strangers | 0.01 [-0.0467 – 0.0670] |
|  | Generic - Work versus Generic - Strangers | 0.09 **[0.0410 – 0.1414]** |
|  |  |  |
| **Narc** | Generic - Family versus Generic - Friends | -0.09 **[-0.1389 – -0.0437]** |
|  | Generic - Family versus Generic - Work | -0.11 **[-0.1622 – -0.0607]** |
|  | Generic - Family versus Generic - Strangers | -0.04 [-0.0982 – 0.0174] |
|  | Generic - Friends versus Generic - Work | -0.02 [-0.0617 – 0.0210] |
|  | Generic - Friends versus Generic - Strangers | 0.05 **[0.0022 – 0.0992]** |
|  | Generic - Work versus Generic - Strangers | 0.07 **[0.0250 – 0.1171]** |
|  |  |  |
| **Psych** | Generic - Family versus Generic - Friends | -0.07 **[-0.1276 – -0.0139]** |
|  | Generic - Family versus Generic - Work | -0.17 **[-0.2293 – -0.1144]** |
|  | Generic - Family versus Generic - Strangers | -0.12 **[-0.1812 – -0.0613]** |
|  | Generic - Friends versus Generic - Work | -0.10 **[-0.1484 – -0.0545]** |
|  | Generic - Friends versus Generic - Strangers | -0.05 **[-0.1010 – -0.0003]** |
|  | Generic - Work versus Generic - Strangers | 0.05 **[0.0093 – 0.0924]** |

*Note*. Confidence intervals for correlation differences were calculated using the R Package cocor (Diedenhofen & Musch, 2015; formula based on Zou, 2007).

Mach = Machiavellianism; Narc = Narcissism; Psych = Psychopathy; CI = Confidence interval; significant differences (CI does not include zero) in bold.

# References

Cabin, R. J., & Mitchell, R. J. (2000). To Bonferroni or not to Bonferroni: When and how are the questions. *Bulletin of the Ecological Society of America*, *81*(3), 246-248. http://www.jstor.org/stable/20168454

Diedenhofen, B., & Musch, J. (2015). Cocor: A comprehensive solution for the statistical
comparison of correlations. *PLoS One*, *10*(4): e0121945. https://doi.org/10.1371/journal.pone.0131499

Dunn, T. J., Baguley, T., & Brunsden, V. (2014). From alpha to omega: A practical solution to the pervasive problem of internal consistency estimation. *British Journal of Psychology*, *105*(3), 399-412. https://doi.org/10.1111/bjop.12046

Jones, D. N., & Paulhus, D. L. (2014). Introducing the short dark triad (SD3) a brief measure of dark personality traits. *Assessment*, *21*(1), 28-41. https://doi.org/10.1177/1073191113514105

Malesza, M., Ostaszewski, P., Büchner, S., & Kaczmarek, M. C. (2019). The adaptation of the Short Dark Triad personality measure–psychometric properties of a German sample. *Current Psychology*, *38*(3), 855-864. https://doi.org/10.1007/s12144-017-9662-0

Revelle, W. (2008). *psych: Procedures for personality and psychological research* (R Package Version 2.1.9). https://rdrr.io/cran/psych/

Rosseel, Y. (2012). Lavaan: An R package for structural equation modeling. *Journal of Statistical Software*, *48*(2), 1–36. https://doi.org/10.18637/jss.v048.i02

Saucier, G., Bel‐Bahar, T., & Fernandez, C. (2007). What modifies the expression of personality tendencies? Defining basic domains of situation variables. *Journal of Personality*, *75*(3), 479-504. https://doi.org/10.1111/j.1467-6494.2007.00446.x

Schroeders, U., & Gnambs, T. (2018). Degrees of freedom in multigroup confirmatory factor analyses. Are models of measurement invariance testing correctly specified? *European Journal of Psychological Assessment*, *36*(1), 105-113. https://doi.org/10.1027/1015-5759/a000500

Schultze, M. (2018). *Stuart: Subtests using algorithmic rummaging techniques* (R Package Version 0.7.3) [Computer software]. https://rdrr.io/cran/stuart/

Schweizer, K. (2010). Some guidelines concerning the modeling of traits and abilities in test construction. *European Journal of Psychological Assessment*, *26,* 1-2. https://doi.org/10.1027/1015-5759/a000001

Vize, C. E., Collison, K. L., Miller, J. D., & Lynam, D. R. (2020). The “core” of the dark triad: A test of competing hypotheses. *Personality Disorders: Theory, Research, and Treatment*, *11*(2), 91–99. https://doi.org/10.1037/per0000386

West, S. G., Finch, J. F., & Curran, P. J. (1995). Structural equation models with non-normal variables: Problems and remedies. In R. Hoyle (Ed.), *Structural equation modeling: Issues and applications* (pp. 56–75). Newbury Park, CA: Sage.

Wrzus, C., Wagner, G. G., & Riediger, M. (2016). Personality-situation transactions from adolescence to old age. *Journal of Personality and Social Psychology*, *110*(5), 782–799. https://doi.org/10.1037/pspp0000054

Zou, G. Y. (2007). Toward using confidence intervals to compare correlations. *Psychological Methods, 12*(4), 399–413. https://doi.org/10.1037/1082-989X.12.4.399

1. For further details of the study see the original publication of Vize et al. (2020). We thank the authors for allowing us to use their data for the current study. [↑](#footnote-ref-1)
2. We estimated a model with all SD3 items to get an impression of the fit of the original version. The analysis shows that our expert-based item selection did not materially impact the factorial validity of the item pool compared to the original version. [↑](#footnote-ref-2)
